# Health Tract, No. 222

**Published:** 1865

**Authors:** 


HEALTH TRACT, No. 222.

### MIND LOST.

"DID you ever thank God that you had not lost your mind?" was the abrupt inquiry of a well-dressed stranger one day not long ago, in one of the busiest streets of great London. The time, place, and circumstances so took the gentleman thus addressed by surprise, that it was a moment or two before he could summon presence of mind enough to hesitatingly respond: "Why, no; I don't think it ever occurred to me in that light." "You ought to, for I have lost mine!" replied the stranger, who, passing hurriedly on, was soon lost in the indiscriminate tide of busy, bustling humanity.

What a calamity! The loss of mind—the loss of all intelligence—except for one fitful moment, as in the case above, and then to relapse into dread vacuity, to come out of it, not again for weeks, and months, and weary years, it may be; no recognition of the beautiful sunlight, the sweet songs of birds, the glittering stars of night! dead to all domestic ties; to the prattle of dear children; to all that binds to home, kindred, and friends; to be always, as the deranged are for a large portion of their time, as in a troubled dream, where ideas are mixed in strange combinations which the intellect, now so weak, is vainly striving to arrange and disentangle and comprehend; trying ever so hard to grasp something that is clear, definite, and tangible, but trying uselessly! Once, when a child, we were shown into a room of an insane asylum, where there were half a dozen women; on the instant of entering, one of them dashed at us like a mad hyena, with seething words hissed through clenched teeth! But the keeper was immediately behind us, and the poor thing slunk away into a dark, distant corner, and cowered down upon the floor, as if preparing to ward off the merciless blows of authority; such being the mode of dealing with the unfortunate demented in those early times. Another one, more gentle, was pointed out as the mother of a family of children, seven in number she said, and then voluntarily began to repeat their names; but she could never remember more than five at a time; then she would begin again to count them on her fingers; at no effort did she repeat the same name twice. She seemed to be painfully feeling for the other two, as if she wanted them to be all present to her mind at once; and we left her still making the vain effort. In the light of these facts, while the reader resolves that it shall be hereafter a frequent subject of thanksgiving to the Merciful One, that he has not lost his mind, let wise and consistent efforts be made to avoid those causes which are known to be capable of producing such irreparable calamities; because, if we bring misfortunes on ourselves, we must bear the crushing curse of the same to life's last and bitter end. Restrain all base passions; keep all the appetites in rational control; harbor no malice; cherish no revenges; brood not over circumstances connected with wounded feelings; if wealth takes wings, go lovingly and trustingly to the great Father of all to "give us this day our daily bread"; and if fair-weather friends forsake, cling the more to Him who sticketh closer than a brother; looking hopefully at the same time, and laboring diligently in good-doing to others, for the reward which awaits the faithful beyond the gloom of the grave. So doing you will never bemoan a "Lost Mind!"

